# Guideline-Directed Medical Therapy in Sepsis Survivors with Left Ventricular Systolic Dysfunction: An Observational Study

**DOI:** 10.3390/jcm14093253

**Published:** 2025-05-07

**Authors:** Thomas Oswald, Samuel Malomo, Thomas Alway, Stanislav Hadjivassilev, Steven Coombs, Susan Ellery, Joon Lee, Claire Phillips, Barbara Philips, Rachael James, David Hildick-Smith, Victoria Parish, Alexander Liu

**Affiliations:** 1 Sussex Cardiac Centre, Royal Sussex County Hospital, Brighton BN2 5BE, UK; thomas.oswald@nhs.net (T.O.); s.malomo@nhs.net (S.M.); t.alway@nhs.net (T.A.); stanislav.hadjivassilev@nhs.net (S.H.); steven.coombs@nhs.net (S.C.); susan.ellery1@nhs.net (S.E.); joon.lee@nhs.net (J.L.); rachael.james3@nhs.net (R.J.); david.hildick-smith@nhs.net (D.H.-S.); victoria.parish@nhs.net (V.P.); 2 Intensive Care Unit, Royal Sussex County Hospital, Brighton BN2 5BE, UK; claire.phillips20@nhs.net (C.P.); barbara.philips@nhs.net (B.P.); 3 Brighton and Sussex Medical School, Brighton BN1 9PX, UK

**Keywords:** sepsis, cardiomyopathy, heart failure, guideline-directed medical therapy, LV dysfunction

## Abstract

**Background:** Sepsis survivors can develop left ventricular systolic dysfunction (LVSD) and heart failure. These patients are often treated with guideline-directed medical therapy (GDMT) known to be effective in patients with non-sepsis-related heart failure. This study sought to assess the use of GDMT on sepsis survivors with LVSD. **Methods:** Sepsis survivors with suspected myocardial injury and/or heart failure diagnosed with LVSD in a UK cardiac centre were retrospectively studied. Clinical and transthoracic echocardiography (TTE) data were recorded and analysed. **Results:** Of the 25 sepsis survivors (age 56 ± 11 years; 52% males), 11 (44%) had LVSD (LVEF < 50%). LV end-diastolic internal diameter (LVIDd) was similar between patients with vs. without LVSD (5.2 ± 0.8 cm vs. 4.7 ± 0.8 cm; *p* = 0.214). Patients with LVSD had significantly greater LV end-systolic internal diameter (LVIDs) than those without LVSD (4.0 ± 1.2 cm vs. 2.8 ± 0.6 cm; *p* = 0.027). Tricuspid annular plane systolic excursion (TAPSE) was similar between the two groups (2.1 ± 0.5 cm vs. 2.2 ± 0.6 cm; *p* = 0.910). Of the 11 patients with LVSD, nine patients underwent repeat TTE scans after 6 months [IQR 3–9], most of whom were taking GDMT. The majority (8/9) of these patients demonstrated LV systolic functional recovery (>5% LVEF increase; mean LVEF improvement 16 ± 11%) after GDMT. Reductions were seen in LVIDd (5.3 ± 0.8 cm to 5.0 ± 0.7 cm) and LVIDs (4.1 ± 1.2 cm to 3.7 ± 0.8 cm) after GDMT, though these changes did not reach statistical significance (both *p* > 0.05). **Conclusions:** GDMT appears beneficial in sepsis survivors with LV dysfunction. This finding should be validated on a larger and multi-centre basis to further affirm the value of medical therapy in post-sepsis heart failure.

## 1. Introduction

Sepsis remains one of the most prolific causes of mortality, leading to around 11 million deaths worldwide each year [1]. Left ventricular systolic dysfunction (LVSD) can occur in approximately a quarter of patients with acute sepsis [1]. Acute sepsis related cardiomyopathy, which is cardiac dysfunction that occurs during the acute septic episode, is thought to be a reversible condition [2,3]. A number of pathophysiological factors are believed to be contributory to the aetiology of acute septic cardiac dysfunction, such as peripheral vasodilatation, increased vascular permeability, and changes in cardiac pre- and after-load conditions resulting from systemic inflammation [4]. The clinical management remains mainly supportive with focus on treating the acute sepsis condition itself [5].

There is increasing evidence to suggest that cardiac dysfunction can occur in survivors of sepsis [6]. A recent pilot study showed that around half of sepsis survivors can have evidence of left ventricular (LV) cavity dilatation and systolic dysfunction, with non-ischaemic patterns of myocardial fibrosis [6]. Although the findings need to be confirmed on a larger study, the evidence does indicate that cardiac dysfunction not only can occur during acute sepsis [7], but also in patients who have recovered from the acute septic event [6]. Given the global prevalence of sepsis and the vast numbers of sepsis survivors, improving our understanding of post-sepsis cardiomyopathy is a clinical priority.

Survivors of sepsis are known to suffer an elevated risk of developing long-term cardiovascular complications such as cardiac dysfunction and heart failure [8,9]. Patients with pre-existing (pre-sepsis) left ventricular systolic dysfunction (LVSD) are more likely to develop severe sepsis, with a worse short-term and long-term clinical prognosis [10]. However, in patients without a known history of pre-existing LVSD or heart failure, the mechanism for the development of sepsis-related LVSD remains unclear. Further, the clinical management of sepsis survivors with cardiac dysfunction has limited evidence base. Improving our understanding of the management of post-sepsis cardiac dysfunction is therefore an important priority.

Guideline-directed medical therapy (GDMT) for heart failure forms the first-line treatment for patients with LVSD [11]. GDMT includes the major “pillars” of heart failure therapy, such as beta-blockers, angiotensin-converting enzyme inhibitors (ACE-Is), angiotensin receptor blockers (ARBs) or angiotensin receptor–neprilysin inhibitors (ARNIs), mineralocorticoid receptor antagonists (MRAs), and sodium–glucose co-transporter-2 inhibitors (SGLT2-inhibitors) [11]. GDMT has established clinical evidence for improving the prognosis of patients with heart failure and LVSD [11]. GDMT can lead to improvement in LV systolic function and, in certain cases, normalisation of LVEF [11]. Observational evidence of GDMT use in sepsis survivors with LVSD remains limited, which is important to demonstrate the value of these therapies in post-sepsis heart failure. This study sought to assess the use of GDMT in sepsis survivors with LVSD.

## 2. Methods

### 2.1. Study Subjects and Clinical Data Collection

Consecutive sepsis survivors aged 18 years or over with suspected heart failure (by clinical assessment either during hospital admission or on an outpatient basis) who underwent cardiac function assessments with echocardiography between March 2014 and September 2024 at the Royal Sussex County Hospital, Brighton (a UK tertiary cardiac centre) were included. A total of 25 patients were found to be suitable for the study and were included. This sample size is on a similar scale to other studies performed on acute sepsis patients using cardiac imaging [12,13] and a similar recent study in post-sepsis patients [6].

### 2.2. Ethical Approval

This retrospective study was reviewed and approved by the Research and Innovation Department of the University Hospitals Sussex NHS Foundation Trust and informed patient consent was waived.

### 2.3. Clinical Data Collection

Clinical patient data were collected from the electronic medical records. These data included demographics information, cardiac symptoms, patient co-morbidities, and cardiac medications. The data collected were independently validated by a second observer as referenced to the electronic medical records.

### 2.4. Transthoracic Echocardiography (TTE)

Two-dimensional TTE images, colour flow, and Doppler images were acquired using commercially available systems. Images were taken in multiple views including the parasternal, apical, and sub-costal views over at least two cardiac cycles. Image acquisition was optimised by adjusting the sector size, depth, and gain settings. LV systolic function was assessed visually or by the Simpson biplane method.

### 2.5. Guideline-Directed Medical Therapy (GDMT)

Data pertaining to GDMT for heart failure for each patient in the study were collected from the electronic patient records. In our hospital, GDMT is introduced as soon as clinically feasible after the diagnosis of LV systolic dysfunction has been made by cardiac imaging. The GDMT is then up-titrated to the maximally tolerated doses by a consultant cardiologist with specialist interest in heart failure, working alongside a team of heart function nurse specialists via a multi-disciplinary team (MDT) approach.

### 2.6. Statistical Analysis

Parametric variables were expressed as mean ± standard deviation. Non-parametric variables were expressed as median [interquartile range]. Parametric data were compared using the Student’s *t*-test, paired where appropriate. Non-parametric data were compared using the Mann–Whitney test. Categorical data were compared using the Fisher’s exact test. *p*-values < 0.05 represented statistical significance. Statistical analysis was performed using commercially available software (MedCalc, version 20.104, Mariakerke, Belgium). The data and results were independently validated by a second observer to ensure accuracy.

## 3. Results

### 3.1. Patient Characteristics of Sepsis Survivors

Of the 25 study patients (mean age 56 ± 11 years; 52% males), pneumonia (36%) was the commonest cause of sepsis (Table 1). Patients required intensive care unit (ICU) admission in 44% of cases and 40% of the patients were managed on the medical wards (Table 1). A minority of patients (20%) required intubation, and a similar proportion (20%) required vasopressor support (Table 1). Patients had elevated peak C-reactive protein levels (247 mg/L [70–317]), peak white cell counts (15.4 × 10^9^/L [10.6–22.8]), and peak high-sensitivity cardiac troponin T (108 ng/L [16–1176]). The remaining patient characteristics, including cardiac symptoms and co-morbidities, are shown in Table 1.

### 3.2. TTE and Medical Therapy in Sepsis Survivors

**Figure 1:**
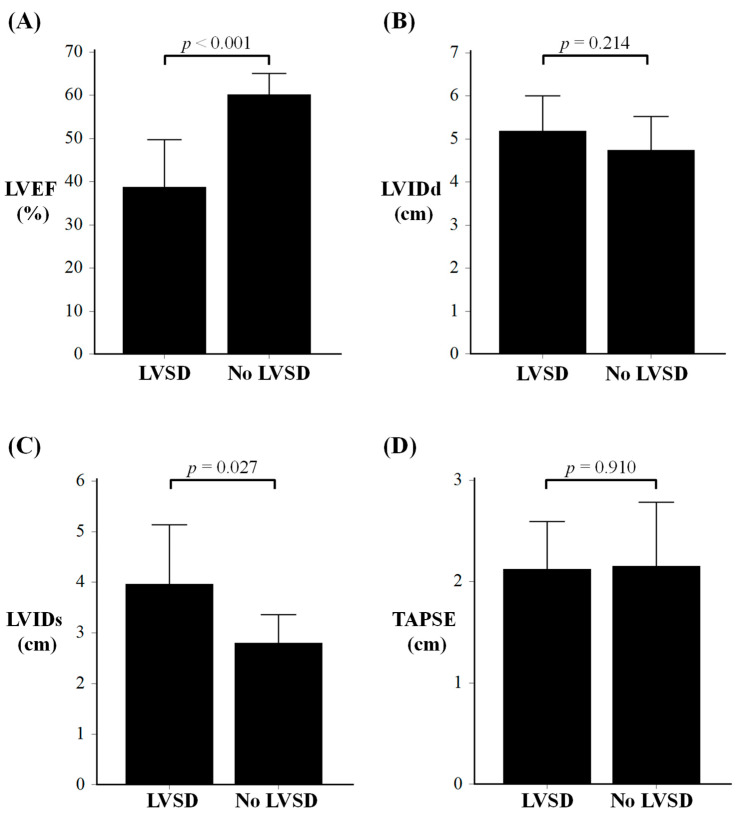
Comparison of echocardiographic findings of sepsis survivors with and without left ventricular systolic dysfunction (LVSD), for LVEF (Panel (**A**)); LVIDd (Panel (**B**)); LVIDs (Panel (**C**)) and TAPSE (Panel (**D**)). LVEF: left ventricular ejection fraction; LVIDd: LV end-diastolic internal diameter; LVIDs: LV end-systolic internal diameter; TAPSE: tricuspid annular plane systolic excursion.

The 25 sepsis survivors in the study underwent their initial TTE assessment a median 10 days (IQR 3–59 days) from the acute sepsis episode. Patients with LVSD (as defined by LVEF < 50%) had similar LV end-diastolic internal diameter (LVIDd) compared to patients without LVSD (as defined by LVEF ≥ 50%); *p* = 0.214 (Table 2 and Figure 1). Patients with LVSD had significantly higher LV end-systolic internal diameter (LVIDs) compared to patients without LVSD; *p* = 0.027 (Table 2 and Figure 1). Both septal and posterior LV wall thickness were similar between the two patient groups; *p* = 0.571 and 0.858, respectively (Table 2). Tricuspid annular plane systolic excursion (TAPSE) and left atrial volume were also similar between patients with vs. without LVSD; *p* = 0.910 and *p* = 0.472, respectively (Table 2 and Figure 1).

Most of the patients with LVSD were taking GDMT, including ACE-Is/ARBs or ARNIs (91%), beta-blockers (82%), and MRA (73%). Over half (55) of patients with LVSD were taking an SGLT2-inhibitor (Table 2). Patients without LVSD took GDMT less frequently (Table 2).

### 3.3. Changes in LVEF in Sepsis Survivors with LV Systolic Dysfunction

**Figure 2:**
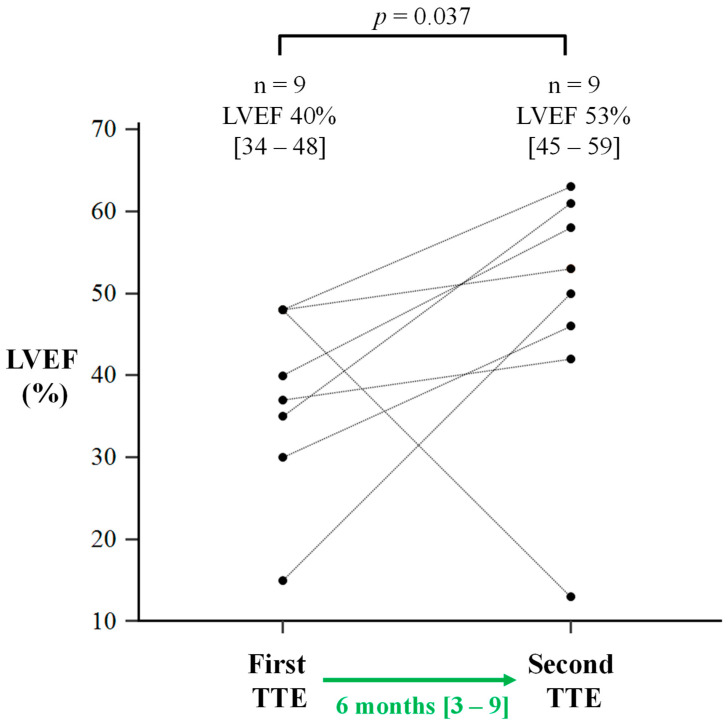
Interval changes in left ventricular ejection fraction (LVEF) in sepsis survivors with LV dysfunction after a period of guideline-directed medical therapy. TTE: transthoracic echocardiogram. Data in square brackets are interquartile ranges, expressed after their respective median values.

Of the nine sepsis survivors with LVSD on TTE who underwent repeat TTE a median of 6 months [IQR 3–9] later, LVEF increased significantly from 40% [IQR 34–48] to 53% [IQR 45–59]; *p* = 0.037 (Figure 2). Regressions were also seen in LVIDd (5.3 ± 0.8 cm to 5.0 ± 0.7 cm; *p* = 0.254) and LVIDs (4.1 ± 1.2 cm to 3.7 ± 0.8 cm; *p* = 0.361), but these changes did not reach statistical significance.

Of the nine sepsis survivors with LVSD who underwent repeat TTE, eight (89%) patients demonstrated significant LV functional improvements (as defined by >5% increase in LVEF; Figure 2).

Patients who demonstrated LV systolic function improvements experienced a mean increase in LVEF of 16 ± 11% (Table 3). Most of these patients were taking GDMT (Table 3). One patient (11%) experienced a significant reduction in LVEF, despite taking an ACE-I, a beta-blocker, and an MRA (Table 3). LVEF improvement was significantly less in patients who required ICU admission (n = 4; LVEF change 5% [IQR -16–5]) compared to patients who did not require ICU admission (n = 5; LVEF change 18% [IQR 16–28]); *p* = 0.013.

## 4. Discussion

This observational study provided an overview of the use of GDMT for heart failure in a single-centre cohort of sepsis survivors with LVSD. The main findings based on this study cohort are as follows: (i) a significant proportion of sepsis survivors suspected of heart failure who underwent TTE have LVSD; (ii) the sepsis survivors with LVSD in this study demonstrated significant LV function improvement several months post-sepsis; and (iii) this LV functional recovery took place in the presence of GDMT in most patients in this study. These findings indicate a potential beneficial role of GDMT in sepsis survivors with LV dysfunction.

### 4.1. Post-Sepsis LV Dysfunction

Sepsis-induced cardiomyopathy has been tissue-characterised using cardiovascular magnetic resonance [12,13]. The acute changes seen during sepsis include myocardial inflammation, oedema, and takotsubo-like contractile dysfunction, which may be reversible [12,13]; however, follow-up studies have been lacking to show whether this hypothesis is true. Autopsy studies have shown that sepsis patients develop myocardial necrosis and interstitial fibrosis, which suggest that the myocardial injury which takes place during the acute septic episode may leave behind myocardial scars [14]. This would suggest that cardiac dysfunction post-sepsis may be driven, at least in part, by pro-fibrotic processes which may be less likely to spontaneously resolve [14].

The results of this study suggest that LV systolic dysfunction may be prevalent in sepsis survivors. This appears in line with existing evidence for the prevalence of sepsis-induced cardiomyopathy reported to occur during the acute septic event [15,16]. Early studies showed that cardiomyopathy which develops during acute sepsis may be a reversible phenomenon shortly after recovery from sepsis [2,3]. However, more recent evidence suggests that LV systolic dysfunction can persist after acute sepsis [6,17], which is in line with the findings of our study. The long-term reversibility of persistent LV dysfunction after sepsis was not previously known and the findings of this study appear to support the notion that LV dysfunction in sepsis survivors is not permanent.

RV systolic dysfunction has been reported in patients with acute sepsis, with variable degrees of reversibility [18]. RV systolic function in the study patients appeared preserved as assessed by TAPSE in sepsis survivors, which may suggest that the deleterious effects of RV dysfunction during the acute episode can recover [7]. This suggestion requires further dedicated investigation since RV function has important prognostic implications in sepsis patients [7].

### 4.2. Role of GDMT in Post-Sepsis LV Dysfunction

In this study, most sepsis survivors with LV systolic dysfunction recovered within a few months whilst on GDMT for heart failure [11]. This supports the notion that GDMT therapy has a potential benefit in these patients, in line with their indications in existing clinical guidelines for the management of non-sepsis-related heart failure [11]. In one patient, the LVEF deteriorated significantly despite the instigation of GDMT. The reason for this anomalous case is unclear, owing to the lack of data from myocardial tissue characterisation methods such as CMR. Further investigation is needed to assess the prevalence of non-responders to GDMT post-sepsis and to assess the underlying aetiology.

The LV function reassessment by TTE was performed at a median 6 months after initial diagnosis of LVSD, which is also in line with existing practice to allow GDMT sufficient time to facilitate LV reverse remodelling [11]. In our cardiac centre, the GDMT therapy was initiated by the treating clinicians (usually the consultant cardiologist) and up-titrated by dedicated heart function specialist nurses who also check for compliance. This multi-disciplinary (MDT)-based practice is in line with clinical guidelines [11]. All but one patient were able to tolerate GDMT which ensured that patients in this study reflected routine clinical care.

The estimation of LVEF was performed by either the Simpson biplane method or visually. Regardless of the method used, clear improvements were recorded in most patients with LV systolic dysfunction who underwent a second (interval) TTE, which reassures that there had been LVEF improvement whilst on GDMT.

Contemporary medical therapies and their uses in heart failure with reduced ejection fraction (HFrEF) continue to evolve, as elegantly summarised by a recent systematic review [19]. Therapies such as ARNIs and SGLT2-inhibitors appear more effective in certain patient populations [19], which are important factors to consider when managing cardiac dysfunction in sepsis survivors. Other therapies, such as vericiguat, although not observed in this study, may also have therapeutic effects in the treatment of post-sepsis cardiac dysfunction, which deserves further investigation.

### 4.3. Limitations and Future Directions

This retrospective observational study has a small sample size, which means that the results may be susceptible to sampling bias. A relatively small study sample size was found on a consecutive basis, which likely reflects the “tip of the iceberg” since post-sepsis cardiac dysfunction is potentially an under-recognised condition. Due to the retrospective nature of the study, invariably some sepsis survivors may have been “missed” who may not have had TTE assessment post-sepsis or were lost to follow-up since post-sepsis may not be as well-known an aetiology as other causes of heart failure. Further, the retrospective nature of the study also naturally does not allow prospective targeted recruitment of such patients which would likely provide more patients over a shorter period available for study. The results will be validated in a larger study, which includes multi-modality cardiac imaging. Not all patients without LVSD had NT-proBNP performed, which would have provided a comparison with patients with LVSD. Further, the follow-up of LVSD patients is performed clinically and guided by LVEF improvement. Serial NT-proBNP was not performed in many patients with LVSD which could also provide additional value in tracking their improvement on GDMT. Owing to ethical reasons and the observational nature of this study, GDMT could not be stopped to assess whether LVSD relapses after LVEF recovery. To help address this issue, a longer follow-up study is ongoing to characterise sepsis survivors with LVEF recovery on GDMT who demonstrate LVSD relapses. Most patients did not undergo more detailed assessment of cardiac structure and tissue characterisation, such as using cardiovascular magnetic resonance (CMR), which may provide further insights into the progression of any myocardial fibrosis. This is the topic of a separate ongoing study. Despite these limitations, this study provided the first association between GDMT and LV function recovery in sepsis survivors and provides an indication of their benefit in this patient cohort.

## 5. Conclusions

GDMT appears beneficial in sepsis survivors with LV dysfunction. This finding should be validated on a larger and multi-centre basis to further affirm the value of medical therapy in post-sepsis heart failure.

## Figures and Tables

**Table 1:** Clinical characteristics of sepsis survivors.

	Sepsis Survivors (n = 25)
Age, years	56 ± 11
Male	13 (52)
BMI, kg/m^2^	28 ± 5
Sepsis cause	
Pneumonia	9 (36)
Unknown origin	3 (12)
Endocarditis	5 (20)
Abscess/soft tissue	5 (20)
Gastrointestinal	2 (8)
Urinary tract infection	1 (4)
Sepsis care setting	
ICU	11 (44)
HDU/CCU	4 (16)
Ward-based care	10 (40)
Sepsis support requirements	
Intubation	5 (20)
Vasopressor	5 (20)
Inotrope	4 (13)
Sepsis peak serum biomarkers	
Peak CRP, mg/L	247 [70–317]
Peak WCC, ×10^9^/L	15.4 [10.6–22.8]
Peak Hs-cTnT, ng/L	108 [16–1176]
Post-sepsis cardiac symptoms	
Chest pain	9 (36)
Dyspnoea	9 (36)
Palpitations	4 (16)
Co-morbidities	
Atrial fibrillation	10 (40)
Smoking (ex-smoker or current)	9 (36)
Hypercholesterolaemia	7 (28)
Hypertension	6 (24)
Diabetes mellitus	5 (20)
COPD/asthma	4 (16)
Ischaemic heart disease	3 (12)
Previous stroke	3 (12)
CKD	1 (4)

BMI: body mass index; CCU: cardiac care unit; CKD: chronic kidney disease; COPD: chronic obstructive airways disease; CRP: C-reactive protein; HDU: high-dependency unit; Hs-cTnT: high-sensitivity cardiac troponin T; ICU: intensive care unit; WCC: white cell count. Continuous data are expressed as mean ± SD or median [interquartile range]. Categorical data were expressed as numbers (%).

**Table 2:** Echocardiographic and medical therapy data in patients stratified by LV systolic dysfunction as defined by LV ejection fraction < 50%.

	LVSD (n = 11)	No LVSD (n = 14)	*p*-Value
Age, years	57 ± 11	55 ± 12	0.696
Echocardiography data			
LVEF, %	39 ± 11	60 ± 5	<0.001
LVIDd, cm	5.2 ± 0.8	4.7 ± 0.8	0.214
LVIDs, cm	4.0 ± 1.2	2.8 ± 0.6	0.027
Septal wall, cm	1.1 ± 0.2	1.1 ± 0.3	0.571
Posterior wall, cm	1.2 ± 0.1	1.2 ± 0.4	0.858
TAPSE, cm	2.1 ± 0.5	2.2 ± 0.6	0.910
LA volume, cm^2^	58 ± 29	71 ± 42	0.472
Medications			
ACE-I/ARB/ARNI	10 (91)	5 (36)	0.012
Beta-blocker	9 (82)	6 (43)	0.099
MRA	8 (73)	0 (0)	<0.001
SGLT-2 inhibitor	6 (55)	1 (7)	0.021
Loop diuretics	5 (45)	1 (7)	0.056
Anti-platelet drugs	5 (45)	5 (36)	0.697
Statin	4 (36)	6 (43)	1.000
Anticoagulation	4 (36)	4 (29)	0.695
NT-proBNP	914 [464–4540]	-	-

ACE-I: angiotensin-converting enzyme inhibitor; ARB: angiotensin receptor blocker; ARNI: angiotensin receptor–neprilysin inhibitor; LA: left atrial; LVEF: left ventricular ejection fraction; LVIDd: LV end-diastolic internal diameter; LVIDs: LV end-systolic internal diameter; LVSD: left ventricular systolic dysfunction; MRA: mineralocorticoid receptor antagonist; NT-proBNP: N-terminal pro B-type natriuretic peptide; SGLT-2: sodium–glucose co-transporter-2; TAPSE: tricuspid annular plane systolic excursion. Continuous data are expressed as mean ± SD or median [interquartile range]. Categorical data are expressed as numbers (%).

**Table 3:** Serial TTE parameter changes in relation to heart failure medical therapy.

	Initial TTE (n = 9)	Repeat TTE (n = 9)	*p*-Value
TTE data			
LVEF, %	40 [34–48]	53 [45–59]	0.037
LVIDd, cm	5.3 ± 0.8	5.0 ± 0.7	0.254
LVIDs, cm	4.1 ± 1.2	3.7 ± 0.8	0.361
Septal wall, cm	1.0 ± 0.1	1.0 ± 0.2	0.798
Posterior wall, cm	1.2 ± 0.1	1.0 ± 0.1	0.007
TAPSE, cm	2.1 ± 0.5	2.1 ± 0.5	0.902
TTE interval, months		6 [3–9]	
LVEF change			
Improvement (>5%)		8 (89)	
LVEF increase, %		16 ± 11	
Deterioration (>5%)		1 (11)	
GDMT			
ACE-I/ARB/ARNI		8 (89)	
Beta-blocker		7 (78)	
MRA		7 (78)	
SGLT2-inhibitor		4 (44)	

ACE-I: angiotensin-converting enzyme inhibitor; ARB: angiotensin receptor blocker; ARNI: angiotensin receptor–neprilysin inhibitor; GDMT: heart failure guideline-directed medical therapy [11]; LVEF: left ventricular ejection fraction; LVIDd: LV end-diastolic internal diameter; LVIDs: LV end-systolic internal diameter; MRA: mineralocorticoid receptor antagonist; SGLT-2: sodium–glucose co-transporter-2; TAPSE: tricuspid annular plane systolic excursion; TTE: transthoracic echocardiography. Continuous data are expressed as mean ± SD or median [interquartile range]. Categorical data are expressed as numbers (%).

## Data Availability

Patient clinical data in the study cannot be publicly shared but an anonymised version can be provided on reasonable request to the corresponding authors.
